# Food for the Sick and How to Prepare It

**Published:** 1900-11

**Authors:** 


Food for the Sick and How to Prepare It. With a Chapter on Food for the Baby. By Edwin Charles French, M. D. Louisville: John P. Morton & Co. 1900. [Price, $1.00.]

This book is one which both general practitioner and nurse will find valuable. It contains very complete diet lists for use in general medicine, nervous diseases, and after surgical operations. A large number of excellent recipes are also included, together with a chapter on infant feeding. The mechanical part of the book reflects credit on the publishers.

